# Supplementary material for: ARIP: A Tool for Precise Interatomic Contact Area and Volume Calculation in Proteins
Source: Int J Mol Sci. 2024 May 9;25(10):5176. doi: 10.3390/ijms25105176 (PMC11120937; doi:10.3390/ijms25105176)
Supplement: Supplementary file 1 [file ijms-25-05176-s001.zip › ijms-2951271-supplementary.pdf]

International Journal of
Molecular Sciences

MDPI

ARIP: a tool for precise interatomic contact area and volume calculation in proteins

Tao Ma [1,2,3,†], Wenhui Li [1,2,3,†], Zhiping Tang [1,2,3], Xiangwei Sun [1,2,3], Lijuan Li [1,2,3], Zhonghua Liu [1,2,3,*] and Gaihua Zhang [1,2,3,*]

1 The National and Local Joint Engineering Laboratory of Animal Peptide Drug Development, College of Life Sciences, Hunan Normal University, Changsha 410081, China; xiangtao312@outlook.com (T.M.); whliabcc@gmail.com (W.L.); pingguotang498@gmail.com (Z.T.); xiangsun023@gmail.com (X.S.); ligs0116@gmail.com (L.L.)

2 Peptide and Small Molecule Drug R&D Platform, Furong Laboratory, Hunan Normal University, Changsha 410081, China

3 Institute of Interdisciplinary Studies, Hunan Normal University, Changsha 410081, China

* Correspondence: liuzh@hunnu.edu.cn (Z.L.); ghzhang@hunnu.edu.cn (G.Z.)

† These authors contributed equally to this work.

Supplemental Figures and Tables

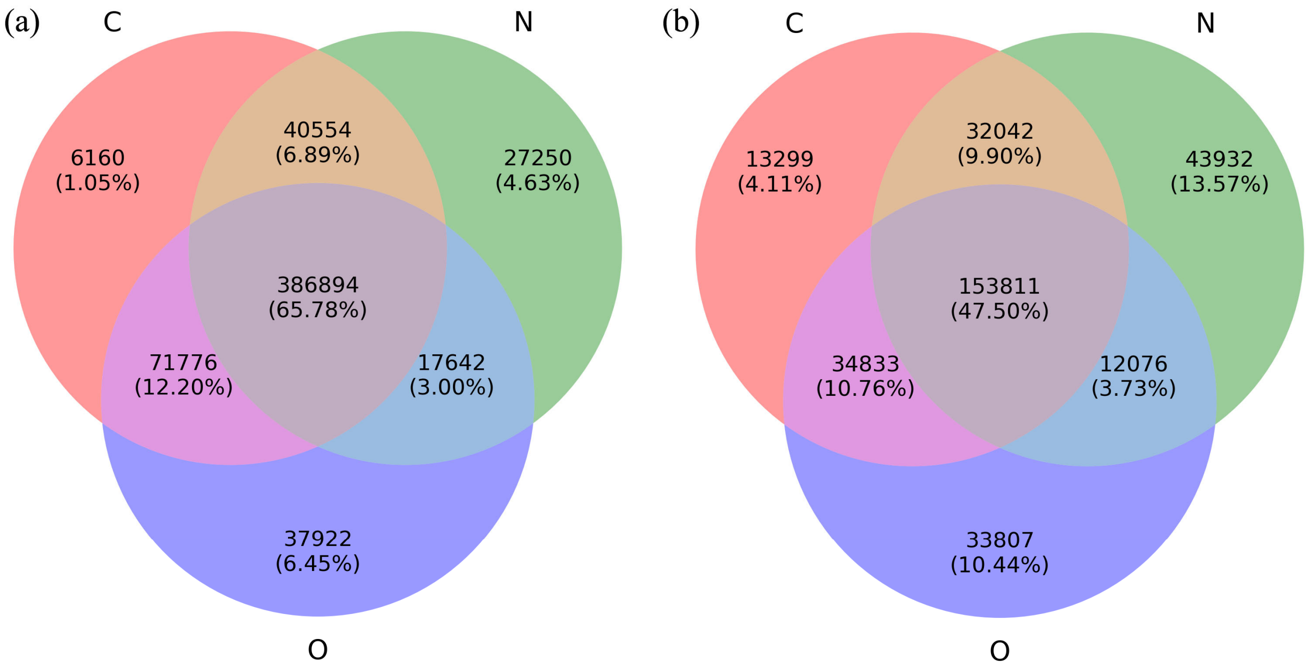

Figure S1. The number of nearest water molecules to the "C", "N" and "O" atoms of the peptide plane without (a) or with interference (b) from other hydrophilic atoms.

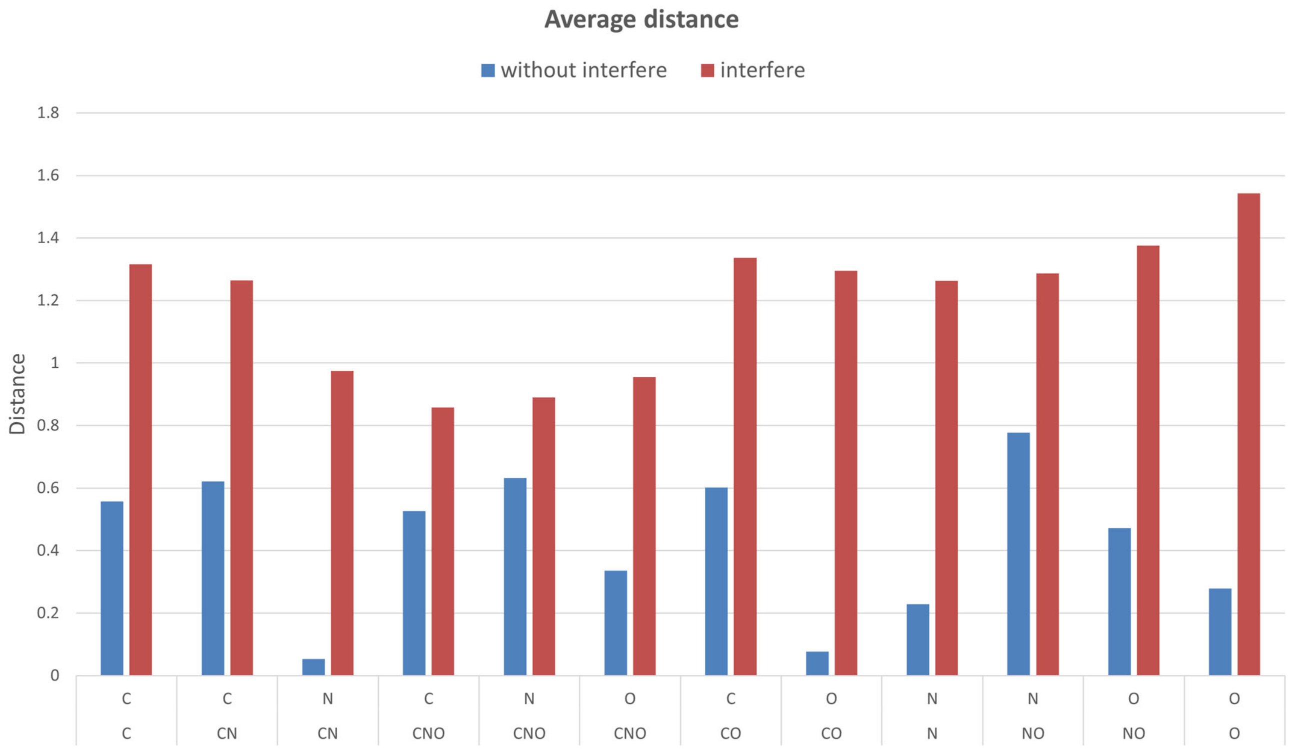

Figure S2. The average distance (Å) of the nearest water molecules to the "C", "N" and "O" atoms of the peptide plane without or with interference from other hydrophilic atoms.

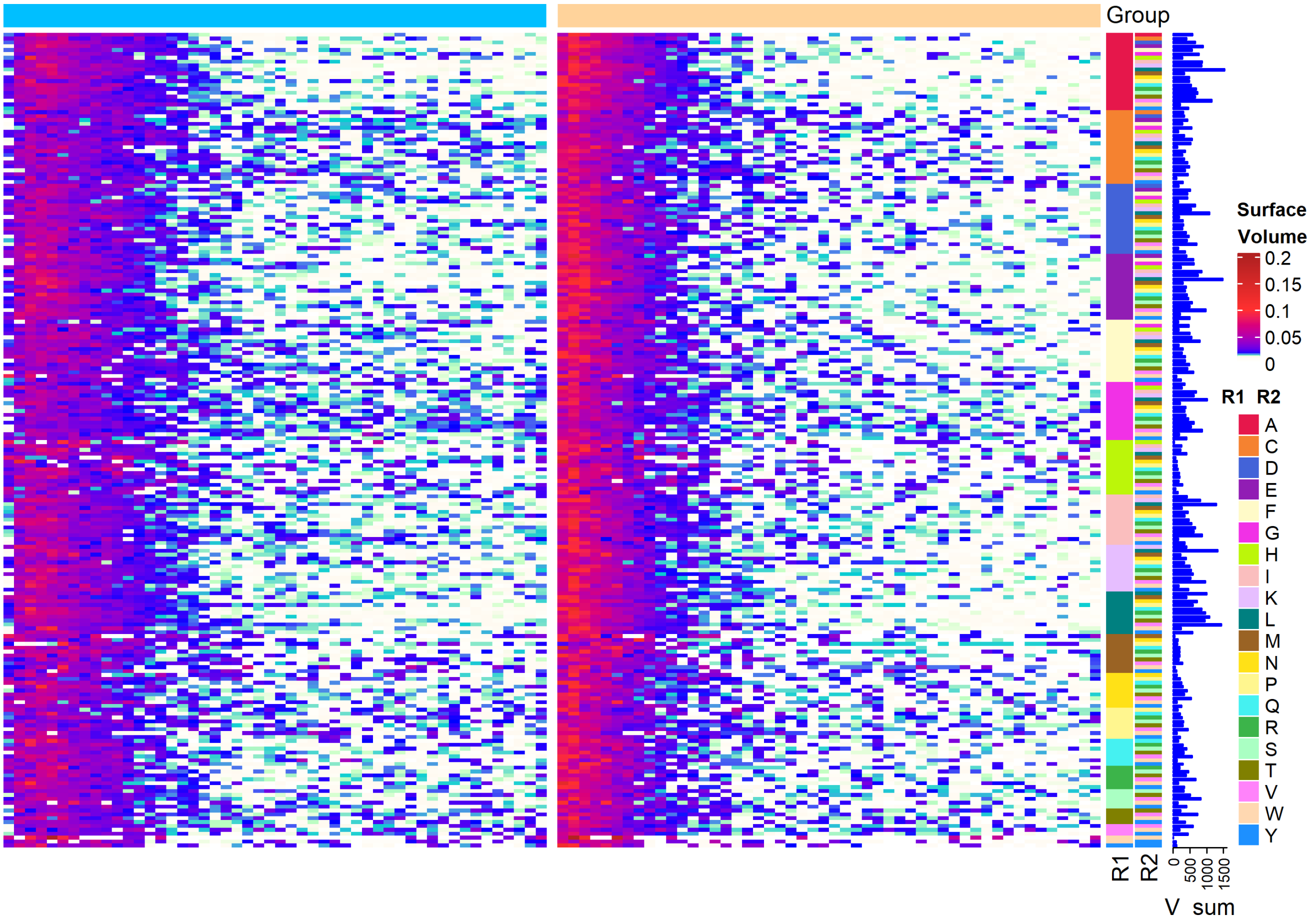

Figure S3. Heatmaps for small contacts area and contacts volume in intra-chain interactions. Values less than 5Å² /Å³ were aggregated using a bin size of 0.1 Å² /Å³ for percentage calculation.

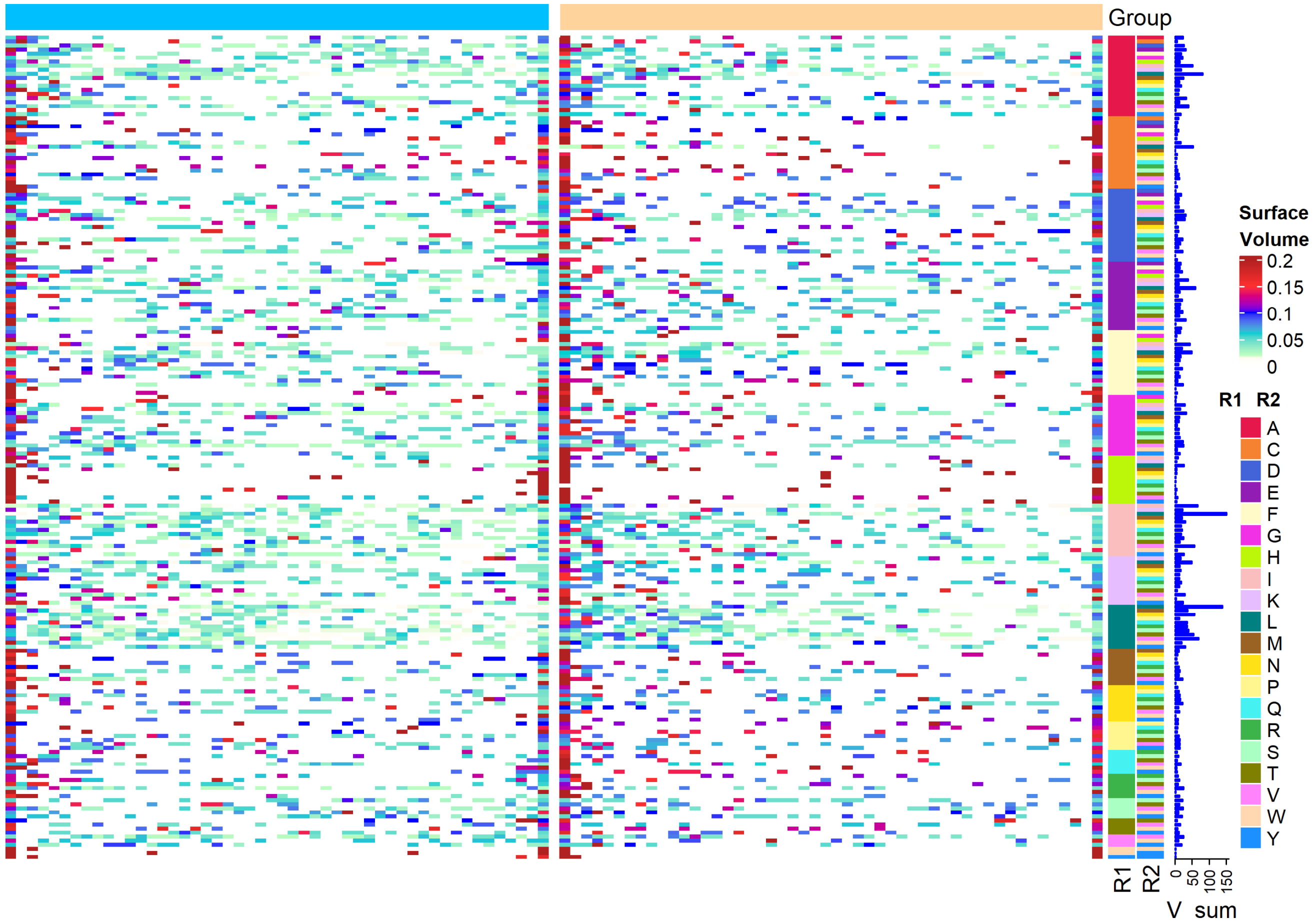

Figure S4. Heatmaps for small contacts area and contacts volume in inter-chain interactions. The number of inter-chain contacts is significantly lower than that in intra-chain interactions. Interestingly, the percentage distributions in inter-chain interactions exhibit a high degree of similarity between contact areas and contact volumes.

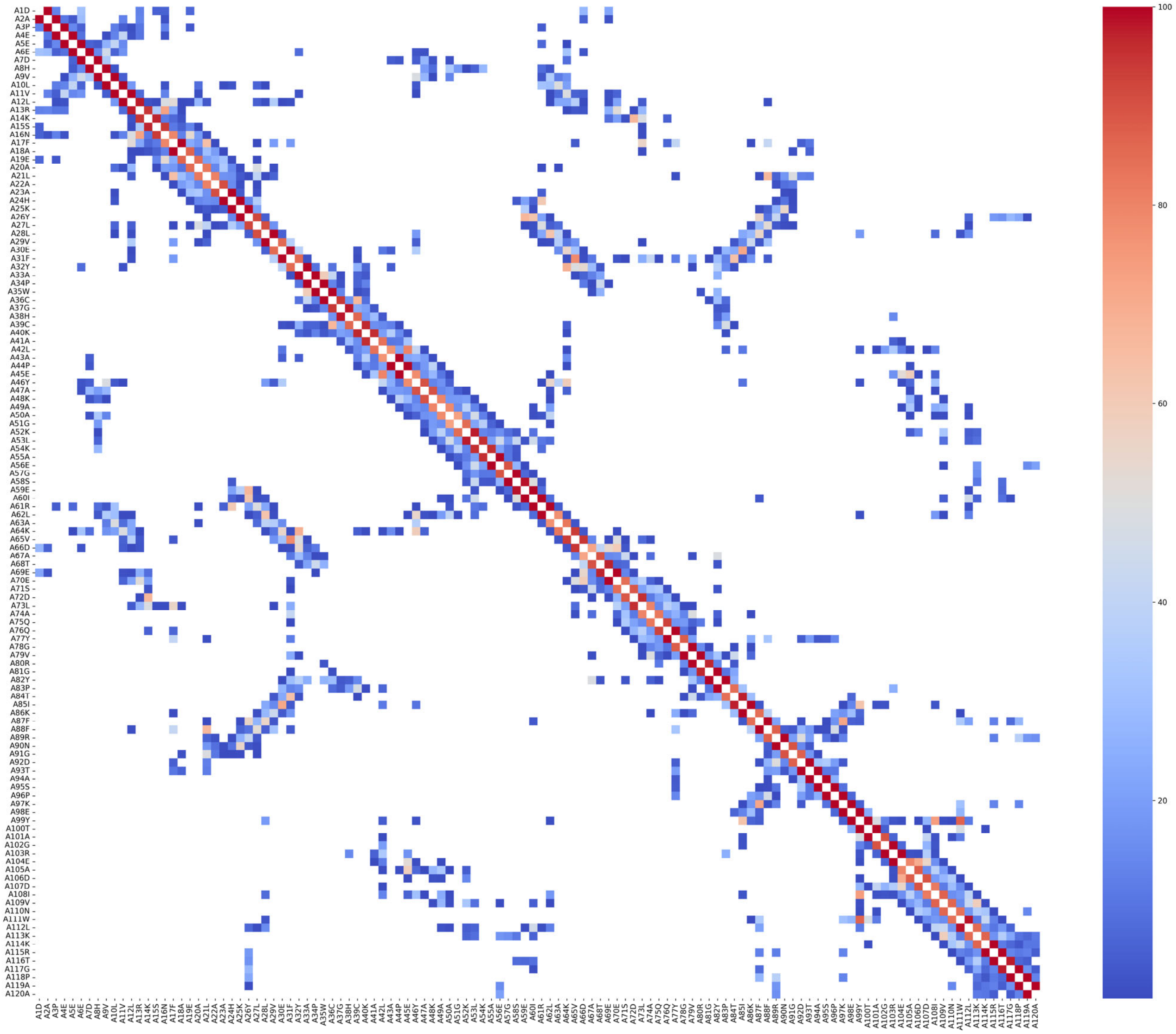

Figure S5. The average contacted volume of residue-residue contacts in all 1MEK models (the maximum volume value is anchoring as 100Å^3 here without using the "-p" switch).

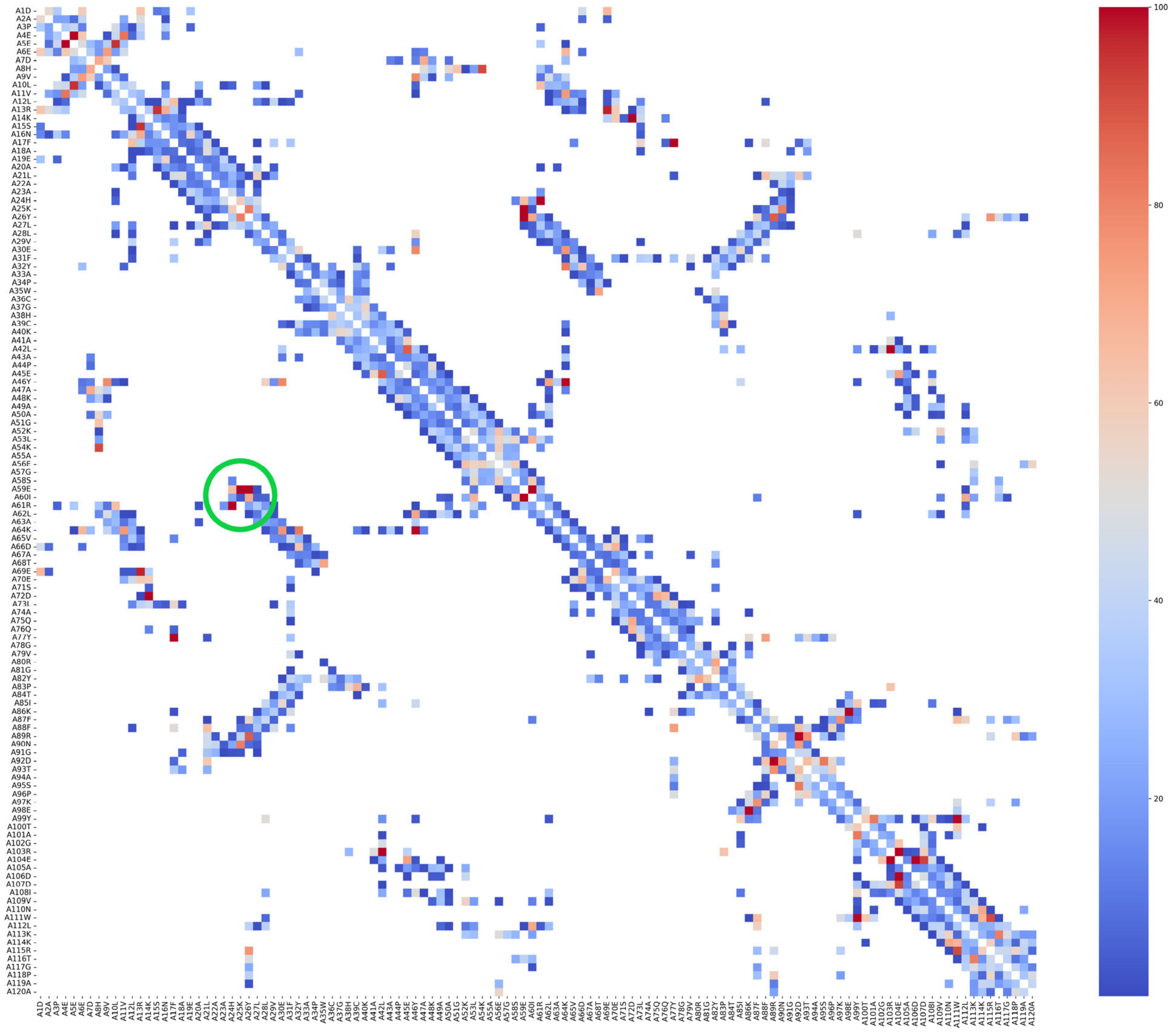

Figure S6. The range (Max-Min) of residue-residue contacted volumes in all 1MEK models.

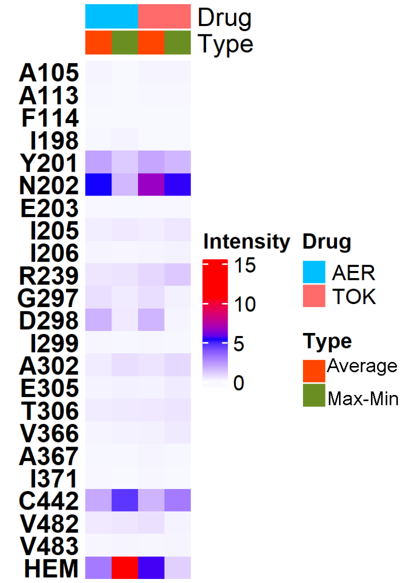

Figure S7. The average and the range (Max-Min) of the interaction volume of CYP17A1 to abiraterone (AER) and galeterone (TOK).

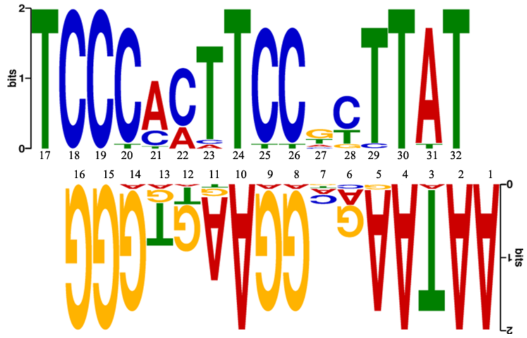

Figure S8. Motif recognition of the DNA alignments of the 22 DNA sequences from SPI1-DNA complexes with MEM v5.5.5

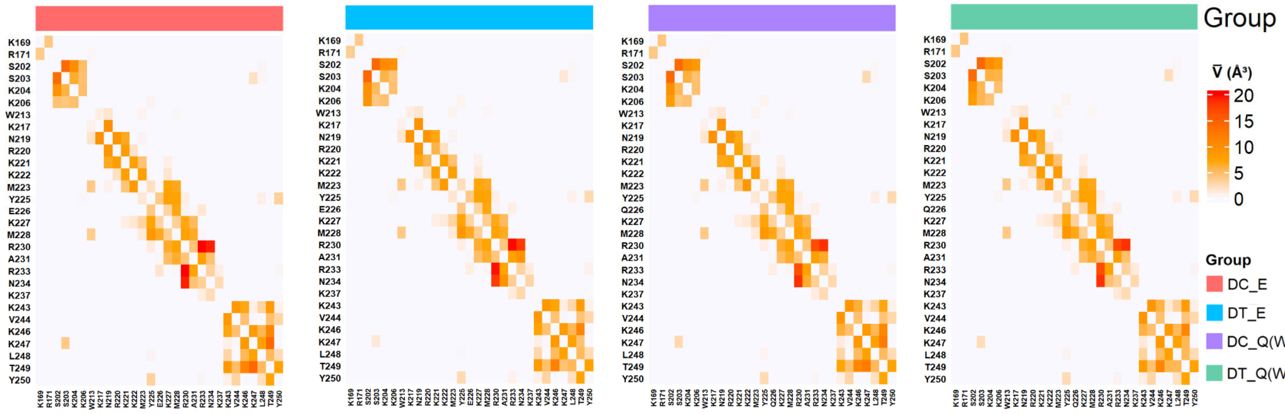

Figure S9. The average of interaction volume of the residue-residue contact in the four groups of SPI1-DNA complexes.

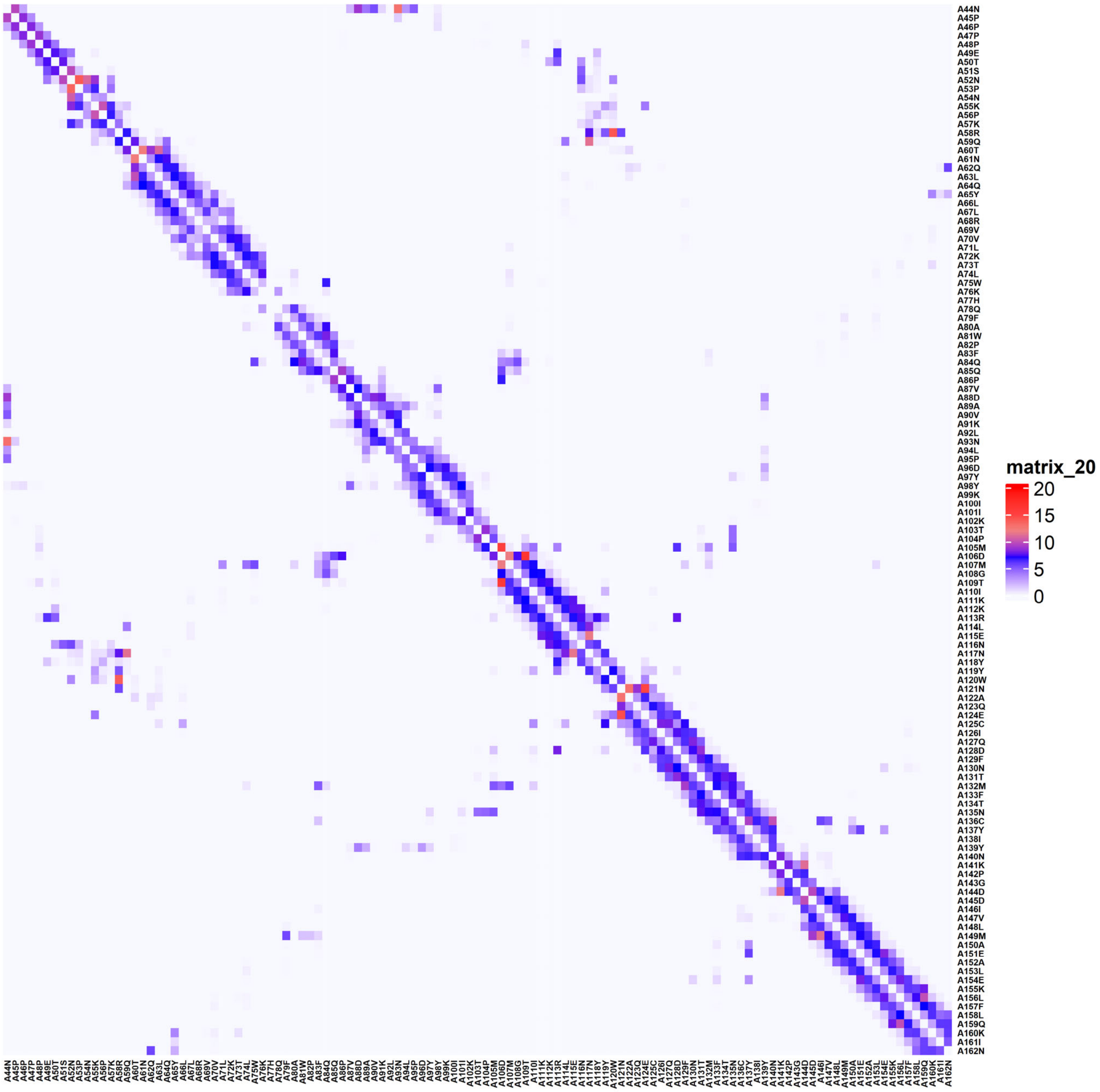

Figure S10. The average interaction volume of residue-residue contacts of 4 states of BRBD4.

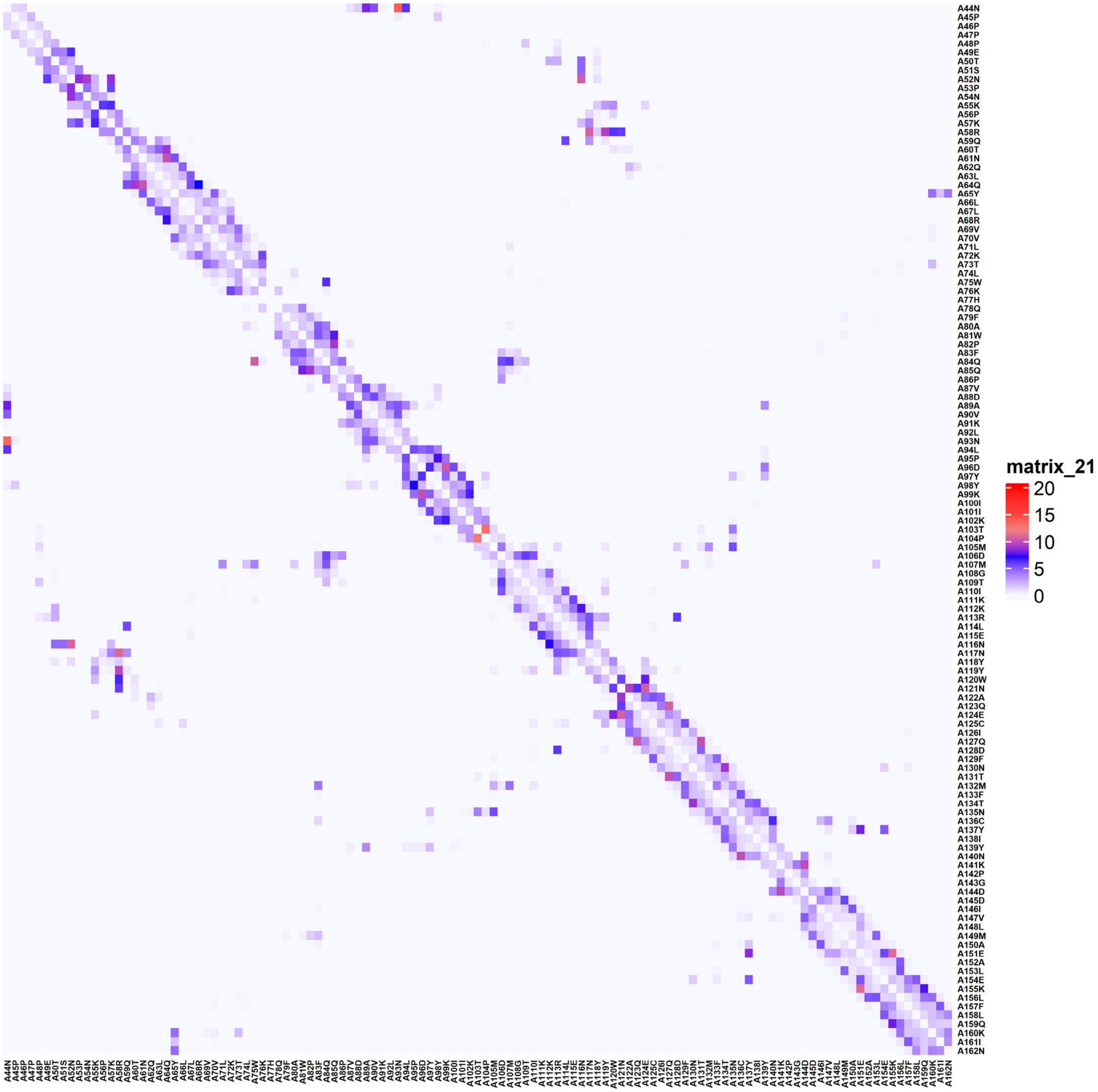

Figure S11. The range (Max-Min) of residue-residue contact volumes of 4 states of BRBD4.

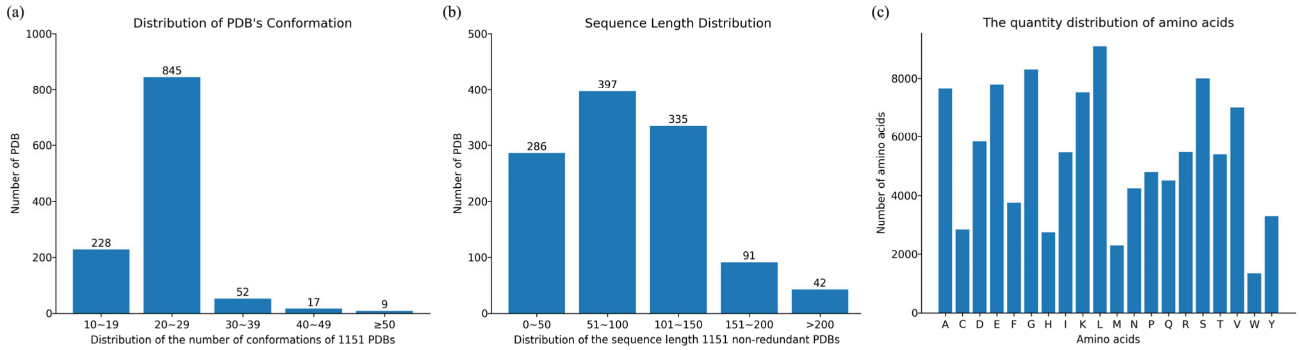

Figure S12. Features of the 1,151 structures solved using NMR technology.

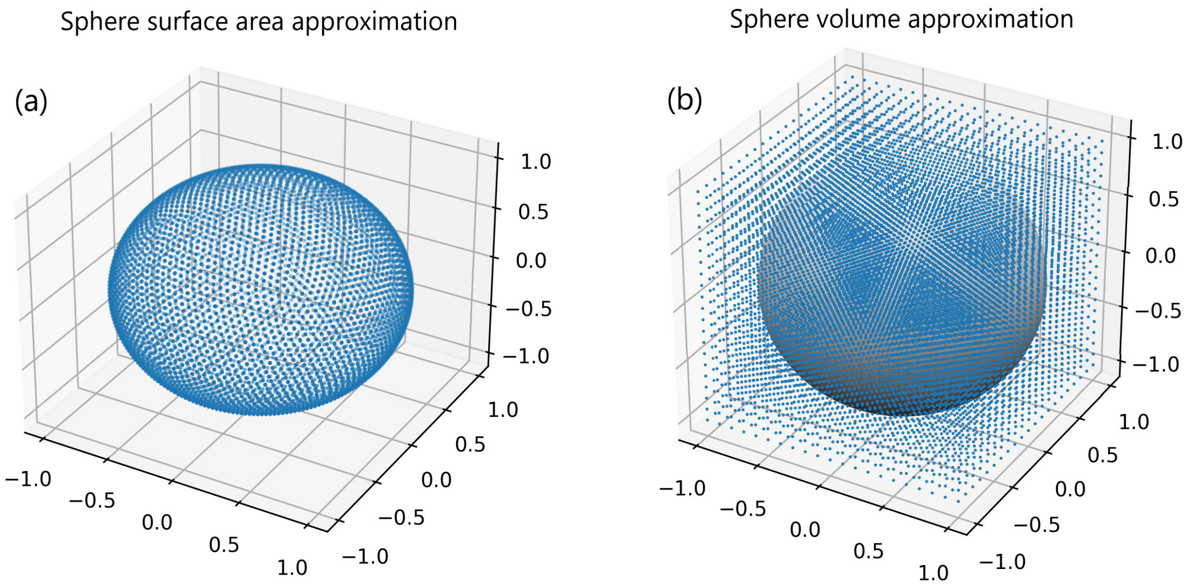

Figure S13. (**a**) Sphere surface area approximation. This demonstrates a sphere shell uniformly divided into 1000 grids, each representing a small area; (**b**) Sphere volume approximation. The volume of the sphere is divided by cubic grids in three-dimensional space.

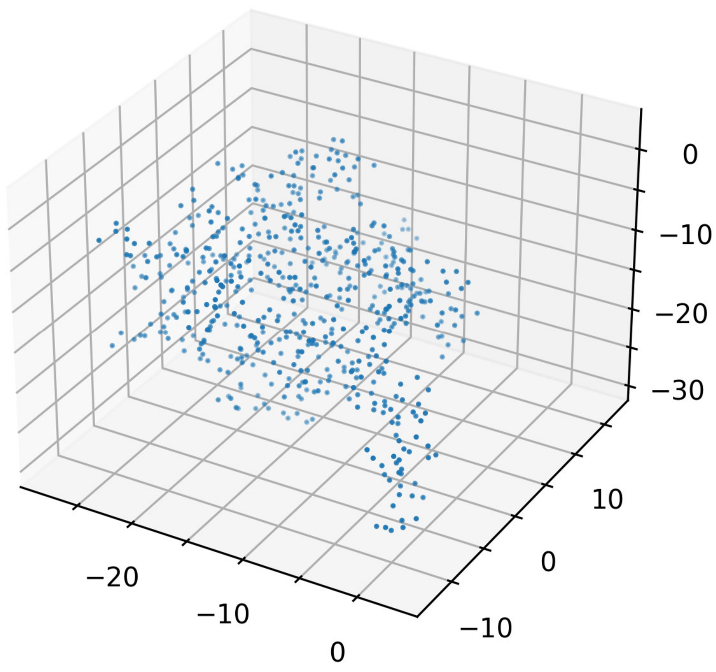

Figure S14. Simple visualization for heavy atoms in PDB 1A0N (MODEL 1).

Table S1. PDBs used for performance comparison.

X-ray diffraction PDB ID	Solution NMR PDB ID
1HJE, 1EJG, 1IUA, 1R6J, 1UCS, 1FY5, 1FY4, 1GDN, 1FN8, 1PQ7, 1YK4, 2DSX, 1GCI, 1W0N, 1X6Z, 2IZQ, 2B97, 2PVE, 2VB1, 1US0, 2WFI, 3AL1, 3A38, 3A39, 2I16, 2I17, 3NIR, 2WFJ, 2QXW, 3MFJ, 4G13, 3MI4, 4HP2, 3X34, 3X2M, 3BCJ, 2IXT, 3W5H, 4I8H, 4I8G, 4NAG, 4M7G, 3D43, 4LB3, 4LB4, 5AL6, 4LBR, 4LBS, 5D8V, 4ZM7, 5OQZ, 5TDA, 5KWM, 5GV7, 5MN1, 5GV8, 6ANN, 5WQQ, 5MNK, 6MW0, 6MW1, 6MW2, 6E6O, 6TOV, 6UF7, 6UF8, 5WQR, 6UFA, 4REK, 4UA6, 6SYJ, 5YCE, 7A5M, 7TLS, 7TLU, 7TME, 7TMH, 7FEZ, 7TMI, 6JGJ, 7KR0, 6ZM8, 7R2H, 6L27, 7WKB, 8C5N, 1DPL, 1CBN, 2O9S, 3X32, 6MVZ, 3X2L, 2JFR, 7ETN, 1N55, 2VXN, 2J8T, 4Y9W, 8C3X, 6MZ2	8B4R, 7YRV, 8B4S, 8BD2, 7XX9, 7XX8, 8AU4, 8A4F, 8BFG, 8BDV, 8BGF, 8BO0, 8BWW, 8ENP, 8CGF, 8CH7, 8EP5, 8ERU, 8F04, 8FD4, 8FA3, 8GUC, 8FKM, 8GQO, 8FG1, 8GXT, 8HR3, 8HVS, 8I53, 8IM5, 8P66, 8PEK, 8POI, 8PWT, 8QHH, 8QHI, 8SED, 8SEM, 8SVB, 8SXM, 8TT7, 8TV4, 8TXS, 8TZM, 8U27, 8ULM, 8UBH, 8WWX, 8BWT, 8HWU

Table S2. PDB groups for protein SPI1 structures.

	Q226	E226
**D28C**	8E3K，8E4H, 8E5Y, 8EBH, 8EJ6, 8EKV, 8ENG, 8EO1, 8EO4	8EK3, 8EMD
**D28T**	8E3R, 8EE9, 8EKJ, 8EQG	8EJ8
